# Endoscopic intranasal orbital decompression in the course of Graves’ orbitopathy

**DOI:** 10.1186/1756-6614-6-S2-A66

**Published:** 2013-04-05

**Authors:** Antoni Krzeski, Mirosław J Szczepański

**Affiliations:** 1 Department of Otolaryngology, Medical University of Warsaw, Warsaw, Poland

## 

The authors present the endoscopic intranasal orbital decompression, one of the surgical techniques used for orbital decompression. It can be applied for various indications. The most common indications are: maxillo-facial trauma, orbital haemorrhage, subperiorbital abscess as the complication of sinusitis, optic neuropathy, thyroid-associated orbitopathy (Graves’ orbitopathy). The authors conclude that the endoscopic transnasal orbital decompression is a valuable and safe operational technique for patients requiring orbital decompression.

